# Chronic Liver Disease in Humans Causes Expansion and Differentiation of Liver Lymphatic Endothelial Cells

**DOI:** 10.3389/fimmu.2019.01036

**Published:** 2019-05-15

**Authors:** Beth A. Jiron Tamburini, Jeffrey M. Finlon, Austin E. Gillen, Michael S. Kriss, Kent A. Riemondy, Rui Fu, Ronald P. Schuyler, Jay R. Hesselberth, Hugo R. Rosen, Matthew A. Burchill

**Affiliations:** ^1^Division of Gastroenterology and Hepatology, Department of Medicine, School of Medicine, University of Colorado, Aurora, CO, United States; ^2^Department of Immunology and Microbiology, School of Medicine, University of Colorado, Aurora, CO, United States; ^3^RNA Biosciences Initiative, School of Medicine, University of Colorado, Aurora, CO, United States; ^4^Department of Biochemistry and Molecular Genetics, School of Medicine, University of Colorado, Aurora, CO, United States; ^5^Keck School of Medicine, University of Southern California, Los Angeles, CA, United States

**Keywords:** lymphatic endothelial cells, cirrhosis, fibrosis, non-alcoholic steatohepatitis, hepatitis C virus, alcoholic liver disease, interleukin-13, oxidized low density lipoprotein

## Abstract

Liver lymphatic vessels support liver function by draining interstitial fluid, cholesterol, fat, and immune cells for surveillance in the liver draining lymph node. Chronic liver disease is associated with increased inflammation and immune cell infiltrate. However, it is currently unknown if or how lymphatic vessels respond to increased inflammation and immune cell infiltrate in the liver during chronic disease. Here we demonstrate that lymphatic vessel abundance increases in patients with chronic liver disease and is associated with areas of fibrosis and immune cell infiltration. Using single-cell mRNA sequencing and multi-spectral immunofluorescence analysis we identified liver lymphatic endothelial cells and found that chronic liver disease results in lymphatic endothelial cells (LECs) that are in active cell cycle with increased expression of CCL21. Additionally, we found that LECs from patients with NASH adopt a transcriptional program associated with increased IL13 signaling. Moreover, we found that oxidized low density lipoprotein, associated with NASH pathogenesis, induced the transcription and protein production of IL13 in LECs both *in vitro* and in a mouse model. Finally, we show that oxidized low density lipoprotein reduced the transcription of *PROX1* and decreased lymphatic stability. Together these data indicate that LECs are active participants in the liver, expanding in an attempt to maintain tissue homeostasis. However, when inflammatory signals, such as oxidized low density lipoprotein are increased, as in NASH, lymphatic function declines and liver homeostasis is impeded.

## Introduction

Deaths from chronic liver disease (CLD) have increased by 31% between the years 2000 and 2015 (1). CLD arises due to chronic inflammation in the liver as a result of a number of environmental insults including viral infection (hepatitis C or B virus-HCV/HBV), alcohol consumption (Alcohol associated liver disease-ALD) and diet-induced obesity (Non-alcoholic steatohepatitis-NASH). Due to the regenerative capacity of the liver, the liver participates in a dynamic process that can result in several rounds of injury and repair. However, chronic injury eventually leads to severe fibrosis, cirrhosis, and the decline of liver function. While removal of the insult causing injury can be effective at reversing liver fibrosis (2), many patients with advanced disease do not improve or ultimately progress to cirrhosis (3, 4). As a result, these people remain at an elevated risk for development of hepatocellular carcinoma despite the removal of chronic insult (5). Limited therapeutic options exist for these patients causing the rates of morbidity and mortality to continue to climb (6).

The lymphatic system transports interstitial fluid (lymph) from the tissue to the circulatory system for removal from the body (7, 8). In addition, lymphatics participate in the acquisition of fat and the formation of chylomicrons in the gut (9), reverse cholesterol transport (10), and trafficking of dendritic cells (DCs) from the tissue to the lymph node (LN) (11, 12). During CLD, increased lymphatic permeability has been implicated in the formation of ascites, or fluid accumulation, in the peritoneal cavity (13). Furthermore, increased lymphatic vessel permeability has been demonstrated to increase inflammation and immune dysfunction in other tissues (14–17). Obesity and hypercholesterolemia are also associated with lymphatic permeability, hyperplasia, and inflammation at peripheral sites in humans (18, 19) and in animals (20).

Lymphatic vessels are comprised of lymphatic endothelial cells (LECs). LEC interactions with immune cells can guide trafficking of immune cells as well as promote self-tolerance and enhance protective immunity (21–25). Despite the multi-faceted role of LECs in programming immune responses in the lymph node and skin, the role of lymphatics in coordinating the immune response in the liver has not been addressed. Furthermore, with the advent of single cell sequencing, several reports have addressed different cell populations within the liver, including specific interrogation of liver endothelial cell populations (26–28). However, in none of these reports have lymphatic endothelial cells been identified. Thus, the transcriptional profile and function of liver lymphatic endothelial cells in homeostasis or disease is yet unknown. However, previous case reports from almost 20 years ago, using common endothelial markers and histology, did demonstrate that lymphatic vessels increase in diameter and abundance during chronic viral hepatitis (29, 30). Despite these observations, little to nothing is known about lymphatics in non-viral cases of CLD, or the cause and/or consequence of lymphatic expansion in the liver. As diet-induced CLD has surpassed chronic viral infection as the leading cause of liver transplantation (31), understanding the role of lymphatics in the liver in people with diet-induced CLD is an important functional process that needs to be addressed.

Here we demonstrate a significant increase in lymphatic vessel density in patients with CLD. We show that in addition to virally induced CLD, that non-virally induced CLD also results in a significant increase in lymphatic vessel density in the liver. To identify specific differences in LECs during disease we performed transcriptional profiling of LECs, using a single-cell platform, from non-diseased and diseased human livers. While other endothelial cell populations have been identified by single cell RNA sequencing in the liver, this is the first demonstration of isolation of LECs and subsequent transcriptional profiling of this rare cell population in the liver. We find that LECs from NASH or HCV infected livers engage a transcriptional program that results in more LECs in active cell cycle and more CCL21 expression. However, when comparing the transcriptional profile of LECs from patients with NASH to patients with HCV we find that NASH specifically induces the activation of the IL13 pathway. Furthermore, we demonstrate that not only is the IL13 pathway increased in patients with NASH, but also that oxidized LDL, commonly associated with inflammation in NASH, can induce the upregulation of *IL13* transcript and protein in LECs. Finally, we provide evidence that IL13 expression by LECs occurs specifically in the liver and that oxidized LDL results in the downregulation of the LEC transcription factor, *PROX1*, and reduced lymphatic stability.

## Materials and Methods

### Patient Samples

For immunohistochemistry archived patient specimens were obtained from the University of Colorado Anschutz Medical Campus biorepository core facility (Supplementary Table 1). For single cell sequencing, patients were selected from a biorepository of patients who had undergone liver transplantation and collected under the IRB protocol of HRR and MSK. Transplanted livers were harvested and non-parenchymal cells (NPCs) were isolated and frozen in a single cell suspension as described (32). Additionally, non-diseased NPCs were purchased from Triangle research laboratories (Lonza, Triangle research park, NC). For Non-diseased patients (*n* = 6) the age range was 37 to 58 with a mean age of 49 where five patients were male and one was female. For HCV samples (*n* = 3) the age range was 47–55 with a mean age of 51 and all patients were male. For NASH samples (*n* = 2) the age range was 49–55 with a mean age of 52 and all patients were females. For Ki67 analysis: the age range was 35–61 for diseased patients and included NASH, ALD, HCV, AIH, PBC, and AIH with NASH. The average age of all diseased patients was 49.4 with 62.5% of the patients being female. The same non-diseased patients from Lonza were used as described above. All patients provided written and informed consent and the study was approved by the institutional review boards at the University of Colorado—Anschutz.

### Flow Sorting and Flow Cytometric Analysis

To enrich LECs from hepatic NPCs we thawed frozen samples in RPMI containing 10% Human Serum AB (Gemini Bio-products, West Sacramento, CA) and 1% DNASE (MP Biomedicals, Santa Ana, CA). Cells were washed 2x with PBS containing 2% FBS (Atlas Biologicals, Fort Collins, CO) and stained with antibodies against CD45 (clone HI30), CD31 (PECAM1, clone WM59), Podoplanin (clone NC-08) and CD146 (clone P1H12) from Biolegend (San Diego, CA), and CD68 (clone KP1) from abcam (San Francisco, CA). Cells from either Non-diseased, NASH or HCV were sorted using an aria Fusion sorter (BD Biosciences, Franklin lakes, NJ) and enriched LECs were sorted into RPMI containing 50% human serum AB. Enriched LECs from each condition were pooled for single cell sequencing as described below. Flow cytometric analysis of human liver LECs: isolated liver NPCs were stained with Fixable viability dye 510 (BD Biosciences) and stained with the above surface antibodies. Following surface staining cells were fixed and permeabilized (Thermo Fischer, Waltham, MA) and stained for Ki67 (clone 11F6) (Biolegend). Flow cytometry was performed using a BD FACSCanto II instrument and was acquired with BD FACSDiva software (BD Biosciences). Analysis ws performed using FlowJo 10 (Treestar, Woodburn, OR).

### Single Cell RNA Sequencing

Approximately 10,000 LEC-enriched hepatic NPCs were loaded onto a 10x genomics (San Francisco, CA) controller per manufacturer instructions to generate barcoded single cell GEMs using the 10x genomics 3′ kit. mRNA was converted to cDNA within each barcoded single cell GEM and libraries were generated as previously described. 10x libraries were sequenced using a NovaSeq 6000 (Illumina, San Diego, CA) to a predicted depth of 100,000 reads/cell. All cell preparation was performed at the University of Colorado Genomics Shared Resource Core. To control for sequencing batch effects, a minimum of three non-diseased samples were included with each diseased capture and sequencing run.

### Quantification of Single Cell RNA Sequencing

scRNA-seq data was processed with the 10x Genomics Cell Ranger Suite for demultiplexing, alignment, assignment of reads to genes, and unique molecular identifier (UMI) deduplication to remove PCR duplicates. Further analysis, including cell clustering, cell type identification, marker gene identification, and differential expression analyses was performed using the R packages Seurat (33) and scran (34). For cell cycle analysis Seurat assigns each cell a score based on its expression of G2/M and S phase markers. These marker sets are anti-correlated in their expression levels, and cells expressing neither are assigned to G1 phase. Cells with fewer than 250 detectable genes or >20% of UMIs derived from mitochondrial genes were excluded from the analysis to eliminate cells with insufficient expression data for clustering and dead cells, respectively (Supplementary Figure 1). Ingenuity pathway analysis (IPA) was performed using IPA software (Qiagen, Venlo, Netherlands). Raw data, count matrices, metadata including cluster assignment are available at the Gene Expression Omnibus under accession GSE129933 (https://www.ncbi.nlm.nih.gov/geo/query/acc.cgi?acc=GSE129933).

### Multispectral Fluorescence Immunohistochemistry and Vectra Analysis

Five micron thick tissue sections were sequentially stained for human PDPN, CD3, CD19, CCL21, and CD68. Slides were dewaxed with xylene, heat treated in pH9 antigen retrieval buffer for 15 min in a pressure cooker, blocked in Antibody (Ab) Diluent (Perkin Elmer, Waltham, MA), incubated for 30 min with the primary Ab, 10 min with horseradish peroxidase (HRP)-conjugated secondary polymer (anti-mouse/anti-rabbit, Perkin Elmer, Waltham, MA), and 10 min with HRP-reactive OPAL fluorescent reagents (Perkin Elmer). Slides were washed between staining steps with PBS 0.01% tween 20 and stripped between each round of staining with heat treatment in antigen retrieval buffer. After the final staining round the slides were stained with spectral DAPI (Perkin Elmer), and coverslipped with Prolong Diamond mounting media (Thermo Fisher, Waltham, MA). Multispectral imaging was preformed using the Vectra 3.0 Automated Quantitative Pathology Imaging System (Perkin Elmer). Whole slide scans were collected using the 10x objective and 10 to 20 regions were selected for multispectral imaging with the 20x objective. The multispectral images were analyzed with inForm software (Perkin Elmer) to unmix adjacent fluorochromes, subtract autofluorescence, segment the tissue into lymphatic vessels and non-lymphatic vessels, segment the cells into nuclear, and membrane compartments, and to phenotype the cells according to morphology and cell marker expression. Lymphatic vessel density was quantified using the Nikon AR software where LVD = vessel area/total area × 100%. Lymphatic vessels with CCL21 staining less 0.036 were classified as CCL21^lo^ or negative while lymphatic vessels with a value >0.036 were classified as CCL21^hi^ or positive using inForm software. For CCL21 analysis three Non-diseased, 5 HCV, and three NASH patient samples were interrogated. For T, B, and macrophage cell analysis three or four non-diseased, 3–5 NASH, and 4 HCV patient samples were quantified. A student's *t*-test was performed where two asterisks represents a *p*-value less than 0.001. Patient data is included in Supplementary Table 1.

### Branch Forming Assay

Branch forming assay was performed as previously described (35, 36). Briefly, a 4.2 mg/ml matrigel (Corning, Tewksbury, MA) pad with either 100 μg/ml oxidized LDL (Alfa Aeser, Ward Hill, MA), 0.25 mM Palmitic Acid (Caymen Chemicals, Ann Arbor, MI, or equivalent amount of DMSO was allowed to solidify for one and a half hours. Fifteen thousand Human Lymphatic Endothelial Cells (PromoCell, Heidelberg, Germany) mixed with 100 μg/ml oxidized LDL, 0.25 mM Palmitic Acid, or equivalent amount of DMSO in endothelial cell growth medium (PromoCell) were placed on top of the matrigel pad. Cells were incubated at 37°C for 21 h, then imaged using a Zeiss microscope, and Axiom camera. For RT-PCR the matrigel was dissolved using ice cold 5 mM EDTA rocking on ice for 1 h. HLECs were pelleted and lysed with RLT buffer, mRNA was extracted using the RNeasy micro kit per manufacturer instructions and cDNA was synthesized using the QuantiTect RT Kit (Qiagen, Venlo, Netherlands) following standard protocols. Quantitative PCR was performed on an Applied Biosystems 7,300 Real-time PCR machine and fold changes in mRNA levels were calculated using the delta-delta CT method. For each gene, all samples were normalized to the average fold change of the vehicle treatment group (DMSO). The following Qiagen QuantiTect primers were used: *GUSB* (QT00046046), *IL13* (QT00000511), *PROX*1 (QT01006670), and *VEGFR3/FLT4* (QT00063637).

### Animal Studies

Six to eight week old C56BL6/J mice were IV injected with 85 μg of unlabeled or DIL or DIO labeled human highly oxidized LDL (Kalen Biochemicals, Germantown, MD) or 100 μg PolyI:C (InvivoGen, San Diego, CA). Following indicated incubation time mice were administered a second dose of stimulant. For *in vivo* BFA experiments, 18 h following the second injection mice were administered 250 μg of Brefeldin A as previously described for analysis of *in vivo* cytokine production by T cells (25, 37). Ninety minutes after Brefeldin A injection mice were euthanized and livers and lymph nodes were processed as described (38) with Brefeldin A in each buffer. For flow cytometric analysis of LEC production of IL13, single cell suspensions of liver and lymph node cells were stained with CD45 (clone 30-F11), CD31 (clone 390), PDPN (clone 8.1.1) from biolegend and CD146 (clone P1H12) and IL13 (clone ebio13A) from ebioscience. Flow cytometry was performed using a BD FACSCanto II instrument and data were acquired with BD FACSDiva software (BD Biosciences) or Cyan ADP and acquired with summit software. Analysis was performed using FlowJo 10 (Treestar). All procedures were approved by the University of Colorado School of Medicine Institutional Animal Care and Use Committee.

### Statistical Analysis

For all graphs with statistical analysis an unpaired student's *t*-test was used evaluate statistical significance between two points using Prism software (GraphPad, San Diego, CA). One asterisk denotes a *p*-value of <0.05, two asterisks denotes a *p*-value <0.01 and three asterisks denotes a *p*-value <0.001. Statistical analysis for single cell RNA sequencing (differential expression testing) was performed using the Wilcoxon Rank-Sum Test implemented in Seurat.

## Results

### Lymphatic Vessel Density Increases in End Stage Liver Disease

**Figure 1:**
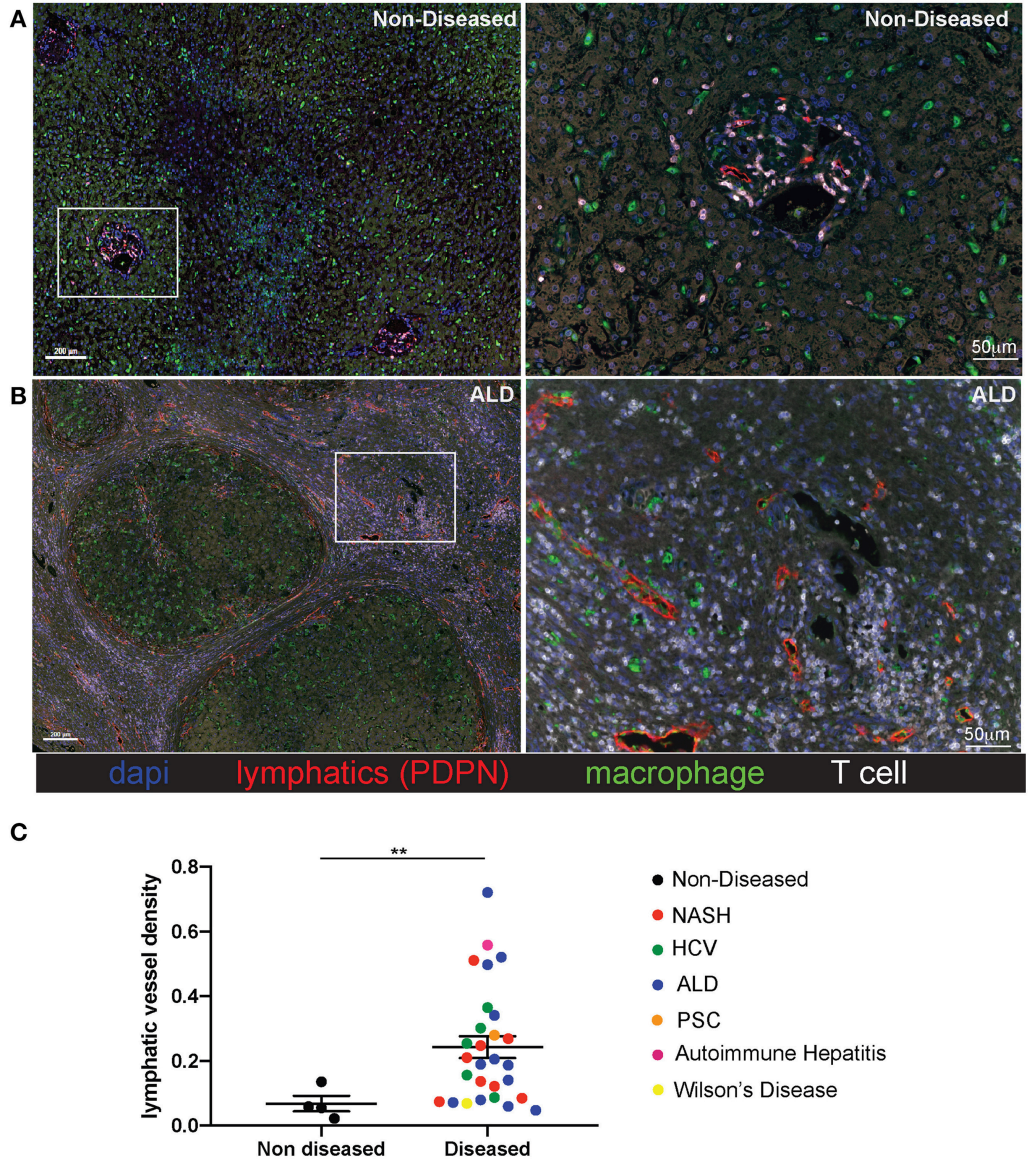
Lymphatic vessels increase in fibrotic regions of cirrhotic livers independent of disease etiology. Liver explants were obtained from cirrhotic patients who received liver transplantation. Non-Alcoholic Steatohepatitis (NASH) (*n* = 8), Alcoholic liver disease (ALD) (*n* = 12), chronic Hepatitis C viral infection (HCV) (*n* = 5), Autoimmune hepatitis (AIH) (*n* = 1), Wilson's disease (*n* = 1), Primary sclerosing cholangitis (PSC) (*n* = 1) and four non-diseased livers. Representative images from non-diseased **(A)** or ALD **(B)** explants are shown. Five micrometer sections were stained with anti-podoplanin (lymphatic vessels D2/40-red), anti-CD3 (T cells-white), anti-CD68 (macrophages-green), and dapi (nuclei-blue) and imaged using the Perkin Elmer Vectra 3.0 imaging system and linear unmixed with inFORM software. **(C)** Lymphatic vessels density was determined using inFORM software and normalized to area in each disease listed and designated by color of dot. Statistical analysis was performed using a student's *t*-test. **P < 0.01.

Liver tissue was obtained from 28 patients with end stage liver disease at the time of transplantation (Non-Alcoholic Steatohepatitis (NASH), Alcoholic liver disease (ALD), chronic Hepatitis C viral infection (HCV), Autoimmune hepatitis (AIH), Wilson's disease, Primary sclerosing cholangitis (PSC), and four patients with non-diseased livers (Supplementary Table 1). Lymphatic vessels in the liver were assessed via immunofluorescence staining with the anti-podoplanin antibody (red) to mark lymphatic vessels in addition to anti-CD3 to mark T cells (white), anti-CD68 to mark macrophages (green), and dapi to label nuclei (blue). Shown are representative images from both a non-diseased liver (Figure 1A) and an ALD patient explant (Figure 1B). In cirrhotic livers, the density of lymphatic vessels is significantly increased, regardless of disease etiology, when compared to liver sections from non-diseased controls (Figure 1C). Changes in LVD were independent of Model for End Stage Liver Disease (MELD) score at time of transplant, age, body mass index (BMI), race, or disease etiology (Supplementary Figure 2). This increased lymphatic vessel density is confined to areas of active inflammation. As such, the lymphatic vessels are in close proximity to regions of inflammation as determined by the frequency of T cells, macrophages, and fibrotic areas. Alternatively, neither lymphatic vessels, nor T cells were found in the regenerative nodules (Figures 1A,B and Supplementary Figure 3). This supports lymphatic expansion as a universal marker and potential critical mechanism for chronic liver disease progression.

### Increased CCL21 Expression and Immune Cell Infiltration Occurs in Cirrhotic Livers

**Figure 2:**
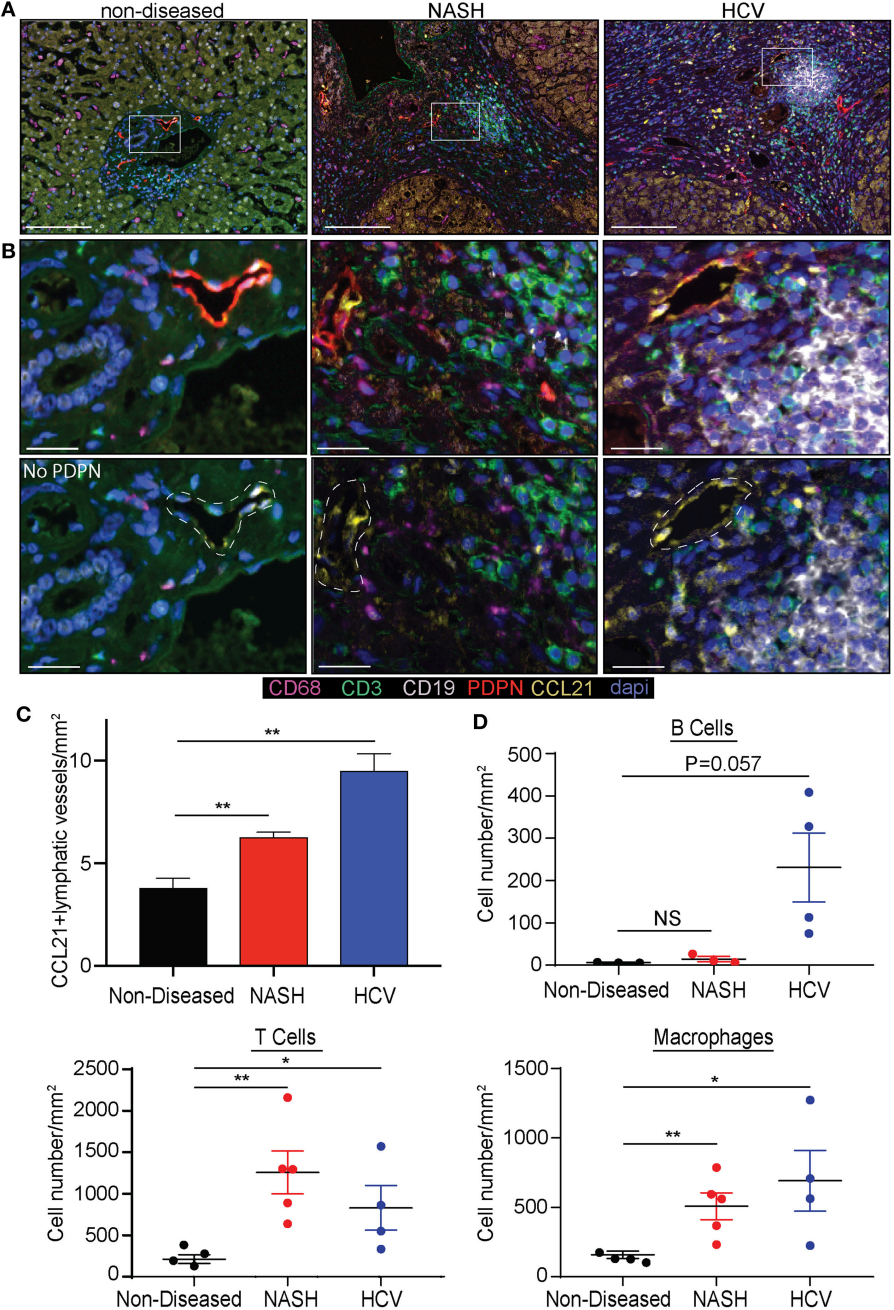
Chronic liver disease induces the expansion of CCL21^+^ lymphatic vessels and immune cell recruitment to the liver. **(A)** Lymphatic vessel (PDPN-Red) expression of CCL21 (Yellow), along with Macrophages (CD68-Magenta), T cells (CD3-Green) and B cells (CD19-White) in non-diseased, NASH, and HCV explanted livers. **(B)** Zoomed in representative examples from **(A)** shown with and without PDPN. White dotted line denotes where lymphatic vessel appears in the image. **(C)** Quantification of CCL21^+^ lymphatic vessels in non-diseased (*n* = 3), HCV (*n* = 5), and NASH (*n* = 3) livers. **(D)** Quantification of B cells, T cells, and Macrophages in liver tissue from Non-diseased (black, *n* = 3), NASH (red, *n* = 3–5) or HCV (blue, *n* = 4). *P < 0.05, **P < 0.01.

As we determined lymphatic vessel density was increased during disease we asked if CCL21, T cell, B cell or macrophage frequency was changed. Others have reported expression of CCL21 by LECs in other tissues and CCL21 is a chemokine that can recruit CCR7+ dendritic cells and T cells (39). We first validated that liver LECs express CCL21 protein using a CCL21 specific antibody. In the imaging of non-diseased and diseased livers we observed that there were populations of LECs that express no or low levels of CCL21 (Figure 2A) while others express high levels of CCL21 protein (Figure 2B). To stratify lymphatic vessels based on CCL21 expression we calculated negative expression to be <0.01 counts, based on a no-CCL21 antibody control, CCL21^lo^ expression to be between 0.01 and 0.036 counts and CCL21^hi^ expression to be between 0.036 and 0.252 counts, as assessed by InFORM software (Supplementary Figure 4). Using PDPN to label lymphatic vessels we found that the number of lymphatic vessels that had CCL21^hi/+^ expression was about 4 vessels per mm^2^ in non-diseased livers, while the frequency of lymphatic vessels with high expression of CCL21 was between 6 and 10 vessels per mm^2^ in patients with HCV and NASH (Figure 2C). These vessels were also often associated with infiltrating immune cells and thus we quantified the accumulation of B cells, T cells and macrophages in the liver. Similar to previous results, we found that end stage liver disease resulted in the significant accumulation of T cells and macrophages in the liver of patients with HCV and NASH while B cells were less frequent (Figure 2D). These studies led us to ask if chronic liver disease induces LECs differentiation in the liver that results in modulation of the inflammatory state of the liver microenvironment.

### Isolation and Single Cell Sequencing of Lymphatic Endothelial Cells in the Liver

**Figure 3:**
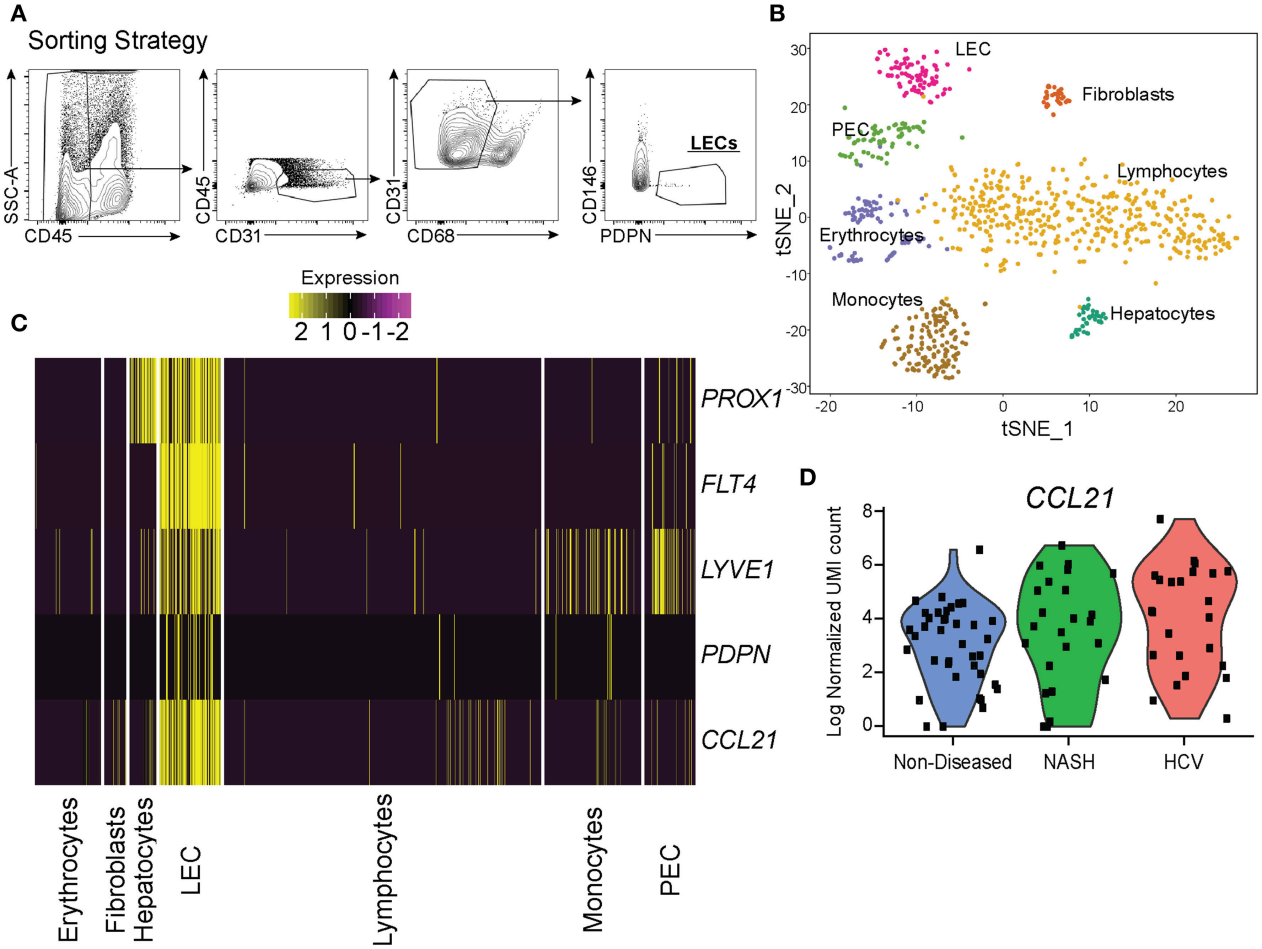
Single cell analysis of liver lymphatic endothelial cells. **(A)** Sorting strategy used to identify LECs. **(B)** TSNE analysis from single cell mRNA sequencing of cells obtained from the sorting strategy in **(A)**. **(C)** Expression of LEC specific genes expressed by isolated cell subsets from the liver (yellow = high, purple = low). **(D)** Normalized CCL21 mRNA expression by LECs from non-diseased (blue), NASH (green), or HCV (red) livers.

To understand how LECs were transcriptionally regulated in the liver we isolated LECs and subjected them to single cell mRNA sequencing. While other endothelial cell populations within the liver have been evaluated transcriptionally, the transcriptome of liver lymphatic vessels has yet to be reported. This could be due to the LECs in the liver being a fragile, rare and difficult to identify population. Therefore, we used our expertise in lymphatic endothelial cell flow cytometry to isolate and acquire the transcriptional signature of lymphatic endothelial cells in the liver. Once LECs from explanted human livers were isolated by flow sorting, using our published liver LEC marker set (38) (Figure 3A), we subjected the sorted cells to single cell mRNA sequencing using the 10x genomics 3′ platform. Individual groups of cells were clustered using TSNE clustering based on transcriptional profile (Figure 3B). Interestingly, while we sorted our population of cells based on known phenotypic markers for LECs we still were able to visualize a number of contaminating cells based on their transcriptional profile. However, using this analysis we were also able to discern that in the non-diseased human liver there are two distinct populations of endothelial cells that are transcriptionally distinct from liver sinusoidal endothelial cells (LSECS) (26).

LECs were identified using the expression of prospero homeobox protein 1 (*PROX1*), lymphatic vessel endothelial hyaluronan receptor 1 (*LYVE-1*), podoplanin (*PDPN*), vascular endothelial growth factor receptor 3 (*FLT4/VEGFR3*), and *CCL21* (Figure 3C). Based on the expression of these LEC-associated markers we were able to divide these two populations into a fully differentiated LEC population and an endothelial cell population that resembles the recently reported portal endothelial cells (PEC) (27). Specifically, these two populations are distinguished by their expression of several markers such as *PROX1, PDPN, CCL21*, Neurotensin, and trefoil factor 3 (*TFF3*) by the LEC cluster; bone marrow stromal antigen two precursor (*BST2*), interferon alpha inducible protein 27 (*IFI27*) and ribonuclease 1 (*RNASE1*) by the other endothelial cluster similar to PECs (Table 1). These factors have been previously reported to be associated with either LECs or blood endothelial cells (BECs) in other model systems and in primary endothelial cell cultures confirming that these subsets are of lymphatic or blood origin, respectively (40, 41). As seen in Figure 2, liver LECs express CCL21 protein while LECs from diseased livers had more CCL21^hi/+^ vessels. This was confirmed by our transcriptional analysis and similar to other reports demonstrating expression of the chemokine *CCL21* by LECs (Figure 3D) (42, 43). We also discovered that *TFF3*—a gene upregulated in hypoxia that induces expression of VEGF and protects barrier function is upregulated by the LEC population (44, 45). The liver PEC-like population has increased expression of genes such as *IFI27* which is also expressed by LECs in the lymph node (46–48); and *HSPG2* encoding Perlecan which is predominantly expressed by BECs, but whose expression can be increased during the final maturation of lymphatic vessels in the skin (49). These data suggest that the PEC-like population may be a progenitor cell for LECs with transcripts found in both cells from blood and lymphatic lineages. Finally, we confirm that the structures we visualized in the livers of cirrhotic patients (Figure 1) are the same LEC population we are evaluating transcriptionally based on the expression of PDPN in this specific endothelial population (Figure 3C). Thus, based on transcriptional profiling we were able to distinguish LECs from other cells in the liver in order to evaluate changes in these cells during chronic liver disease.

**Table 1:** LECs and PECs have similar but distinct transcriptional profiles.

**Gene**	**avg_log2(LEC/PEC)**	**pct.LEC**	**pct.PEC**	**pval**	**Pval adj**
CCL21	5.158128464	0.763	0.045	2.00E-11	2.26E-07
TFF3	3.305995867	0.789	0.045	1.17E-11	1.33E-07
NTS	2.140331515	0.316	0	6.73E-05	0.760564313
ADIRF	1.637091524	0.711	0.227	2.39E-06	0.027008311
S100A10	1.47389027	0.763	0.386	4.70E-06	0.053149996
S100A6	1.29723903	0.789	0.318	1.39E-06	0.015742032
FABP4	1.086755403	0.658	0.091	2.56E-07	0.002893748
JUNB	−1.508928712	0.158	0.636	7.56E-06	0.085490214
AC090498.1	−1.65001069	0.184	0.545	1.62E-04	1
ZFP36	−1.665549178	0.079	0.5	6.20E-05	0.700906334
NBEAL1	−1.699311818	0.105	0.455	2.87E-04	1
RDX	−1.836136132	0.053	0.409	1.89E-04	1
C11orf96	−1.881095457	0	0.273	5.79E-04	1
FCN3	−1.963822515	0.026	0.477	1.15E-05	0.129553478
SAT1	−2.283288374	0.026	0.5	3.07E-06	0.034715003
MTRNR2L12	−2.322539602	0.053	0.523	6.06E-06	0.068538598
PLPP3	−2.351071763	0	0.318	1.66E-04	1
HSPG2	−2.394084071	0.026	0.477	5.88E-06	0.066463691
CCL14	−2.467042053	0.053	0.477	1.60E-05	0.181344818
IFI27	−2.604129481	0.079	0.659	5.81E-08	6.57E-04
BST2	−2.622236874	0	0.432	5.77E-06	0.065268266
RNASE1	−2.767317205	0	0.523	2.99E-07	0.003384829

*Shown is the average log2 gene expression fold change between LEC and PEC in non-diseased livers, the percent of cells in each subset that expresses a given gene, and the raw and adjusted P-values. Data was filtered to include only genes in which the p-value between LEC and PEC was < 0.0006*.

### Liver Disease Results in the Proliferation of Lymphatic Endothelial Cells

**Figure 4:**
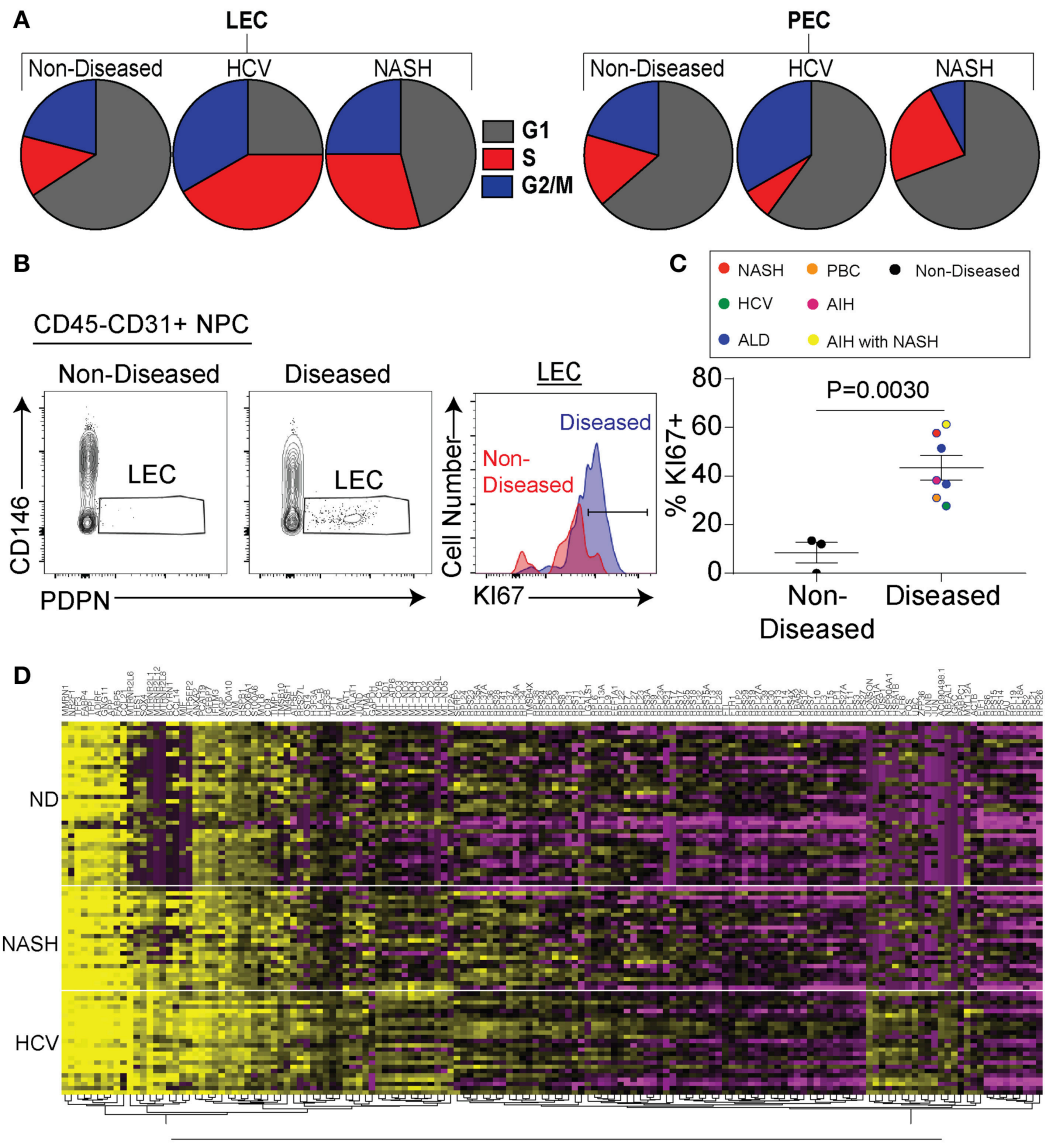
Chronic liver disease results in increased LEC proliferation. **(A)** Frequency of LECs (left) and PECs (right) in each stage of cell cycle based on gene expression from single cell sequencing data. **(B)** Representative flow cytometric profiles of LECs from non-diseased (left and red) or diseased (right and blue). **(C)** Quantification of **(B)**. **(D)** Hierarchical clustering of differentially expressed genes from LECs sorted from non-diseased (ND), NASH, or HCV explanted livers.

As stated above we identified lymphatic endothelial cells in the liver both by flow cytometry and transcriptional profiling. Being that we saw a substantial increase in the frequency of lymphatic vessels in disease (Figure 1) we evaluated if chronic liver disease induced the specific expansion and differentiation of LECs. We first compared liver LECs from non-diseased to diseased livers (both HCV and NASH) and found LECs from diseased livers downregulated pathways involved in apoptosis while upregulating pathways involved in free radical scavenging (Supplementary Table 2). Similarly, upstream pathways activated in LECs from diseased livers included Jun N-terminal kinase (JNK), mitogen-activated protein kinase (MAPK), as well as tumor necrosis factor (TNF) (Supplementary Table 3). We also observed a higher proportion of LECs from diseased patients that are in active cell cycle compared to LECs from non-diseased livers or PECs (Figure 4A). To confirm this transcriptional data we utilized flow cytometry to measure Ki67 expression by LECs from non-diseased and diseased livers. Using this approach, we were able to confirm our transcriptional data demonstrating that a higher frequency of LECs from diseased livers have Ki67 expression compared to LECs from non-diseased livers (Figure 4B). This difference was consistent across patients, suggesting the expansion of LECs is a common event during chronic liver disease (Figure 4C). Finally, when comparing genes between liver LECs from non-diseased, HCV or NASH we found that each group clustered differently (Figure 4D). Many of the same genes were differentially expressed between non-diseased and both NASH and HCV (Figure 4D) suggesting different inflammatory stimuli induce some of the same transcriptional programs. However, while there were many similarities in the transcriptional programs of LECs from HCV or NASH explanted livers there were also differences (Figure 4D). Taken together, these data demonstrate that LECs and PECs, are differentially regulated during chronic liver disease and that chronic liver disease results in the preferential expansion of LECs. These data also suggest that different inflammatory insults may regulate different gene programs in LECs.

### CLD Alters Signaling Pathways in Liver LECs

**Figure 5:**
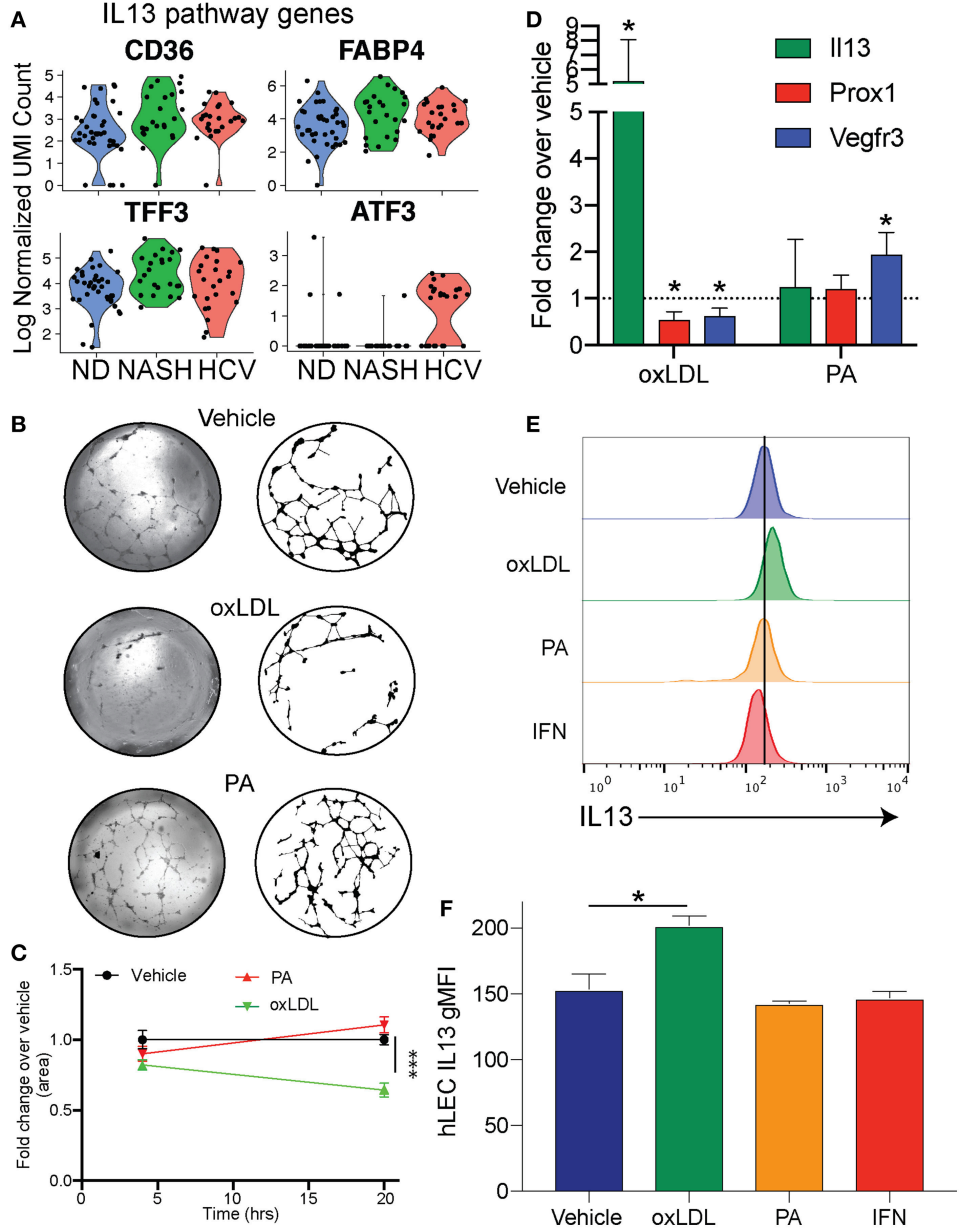
Cholesterol regulates IL13 signaling in LECs. **(A)** Genes involved in IL13 signaling in LECs from Non-diseased (ND, blue), NASH (green), and HCV (red) **(B)**. Representative images of lymphatic branching from hLECs treated for 24 h with Vehicle (DMSO), Ox-LDL (100 μg/ml) or PA (0.25 mM). **(C)** Quantification of **(B)**. **(D)** Quantitative RT-PCR of hLEC treated with the indicated stimulus for 24 h. **(E)** Representative flow cytometric profiles of IL13 protein production by hLECs after 24 h with the indicated stimulus. **(F)** Quantification of **(E)**. *P < 0.05. ***P < 0.001.

To examine differences in gene expression in LECs dependent on disease etiology we compared the gene expression of LECs from NASH patients to HCV patients (Table 2). We also used IPA software to evaluate transcriptional pathways (Supplementary Table 4) induced by the different disease etiologies. Intriguingly, the IL13 signaling pathway was upregulated in LECs isolated from patients with NASH (Supplementary Table 4). IL13 has been shown to be involved both in maintaining lymphatic vessel structure and permeability (35, 50) and in the conversion of hepatic stellate cells (HSC) to myofibroblasts (51, 52). While IL13 expression was not detected in any of the cell types we evaluated, suggesting a signal strength issue; CD36, FABP4 and TFF3, genes that are targets of IL13 signaling, were upregulated (53–55), and ATF3, which inhibits IL13 transcription (56), was downregulated in LECs from NASH livers (Figure 5A). These data not only gave us potential leads to follow, but also led us to the conclusion that while CLD uniformly induces the expansion of lymphatic vessels in the liver, NASH-associated liver disease elicited a unique transcriptional profile in liver LECs that involves IL13 signaling.

**Table 2:** Transcriptional differences between LECs from patients with NASH or chronic HCV infection.

**Gene**	**avg_log2(NASH/HCV)**	**pct.NASH**	**pct.HCV**	**pval**	**pval_adj**
SYTL2	2.154809339	0.5	0.042	2.91E-04	1
AC090498.1	1.266572393	0.875	0.875	1.24E-04	1
EIF1	−0.552823526	0.417	0.958	5.94E-04	1
MALAT1	−0.970590552	0.958	1	9.62E-05	1
DUSP1	−1.06840421	0.333	0.875	4.43E-04	1
IGLC2	−1.268154318	0.125	0.667	4.03E-04	1
MTRNR2L8	−1.483505058	0.125	0.708	2.74E-04	1
DONSON	−1.612257661	0.458	1	1.82E-07	0.002055027
ACVR2B	−1.80158542	0	0.5	9.98E-05	1
IGHG3	−2.106156828	0	0.542	4.18E-05	0.472913317
IGKC	−2.5839658	0.042	0.875	2.81E-08	3.18E-04
MTRNR2L6	−2.796201221	0	0.625	6.66E-06	0.075261418
MTRNR2L1	−3.042838611	0.083	0.875	1.81E-07	0.002050256

*Shown is the average log_2_ gene expression fold change differences between LECs isolated from NASH and HCV, the percent of cells in each subset that expresses a given gene, and the raw and adjusted P-values. Data was filtered to include only genes in which the p-value between HCV and NASH were <0.0006*.

We next asked if these transcriptional differences were a result of direct stimulation of LECs by factors associated with the disease. Therefore, we asked if oxidized LDL (oxLDL) or palmitic acid (PA) affected the lymphatic branching and transcriptional profile of human LECs (hLECs) *in vitro* (Figure 5B). We used oxLDL as it accumulates in response to free radicals generated by inflammation, has been shown to affect other cell types such as macrophages and endothelial cells in atherosclerosis and is elevated in NASH (57–62). Further, oxLDL has a dramatic effect on macrophage activation, while LDL alone does not (63). We used PA as it is an important dietary fatty acid that is largely consumed in foods [reviewed in (64)]. We observed that when LECs were treated with vehicle (DMSO), PA or oxLDL and visualized at 4 and 24 h post-treatment that only the vehicle and PA treated LECs were able to maintain their branched structures over the 24-h period (Figures 5B,C). OxLDL treated LECs were able to form the branch structures, but by 24 h the structures had collapsed (Figures 5B,C). To determine if these dietary constituents were inducing transcriptional changes in the LECs we performed qRT-PCR on the hLECs and evaluated the transcript abundance of *IL13* (Figure 5D) because the IL13 pathway was upregulated in LECs from people with end stage NASH (Figure 5A) and IL13 has been shown to cause defects in lymphatic branching and inhibit *PROX1* expression (35). Interestingly, we found that *IL13* was upregulated only in LECs that were treated with oxLDL, but not PA (Figure 5D). Consistent with LEC structure collapse, oxLDL treatment resulted in the decreased expression of *PROX1* and the *PROX1*-dependent gene, *FLT4 (VEGFR3)* (Figure 5D). This is in contrast to PA which did not affect *PROX1* expression. We next asked if IL13 protein levels were increased after oxLDL, PA or interferon alpha. Interferon signaling is significantly increased in HCV while IL13 signaling is not. We found using flow cytometry that there is a significant increase in protein expression of IL13 in hLECs treated with oxLDL, but not PA or IFN (Figures 5E,F). These data demonstrate that oxLDL can induce transcriptional and functional changes in LECs *in vitro* that are similar to the signaling pathways we observed in patients with NASH. Thus, IL13 signaling in LECs from patients with NASH could be caused by increased levels of oxLDL in the liver.

### OxLDL Uniquely Induces IL13 Production by Liver LECs *in vivo*

**Figure 6:**
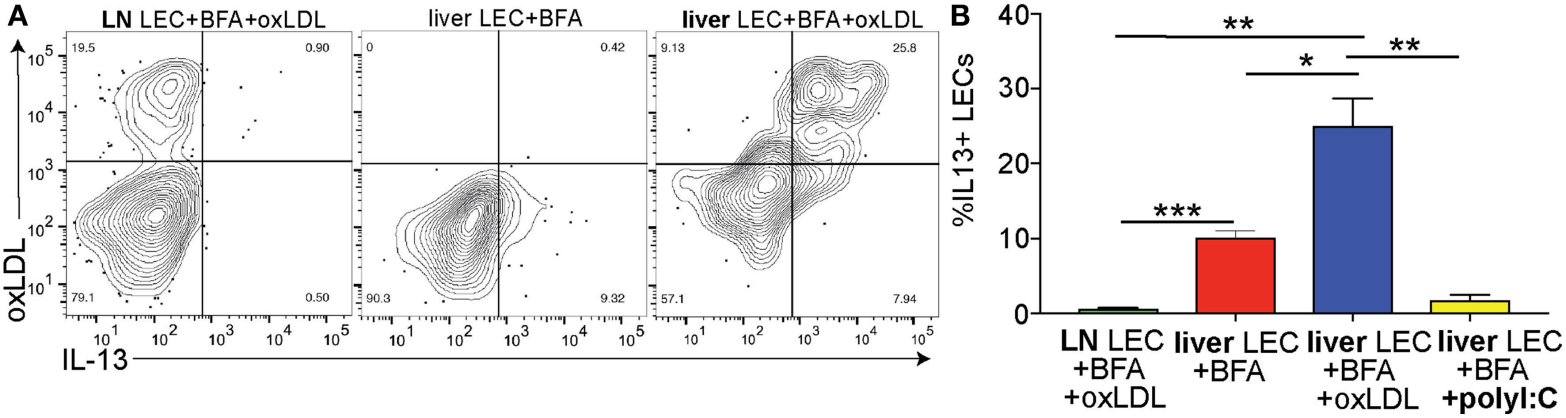
Cholesterol results in IL13 production by liver LECs *in vivo*. **(A)** Representative flow plots of cholesterol uptake and IL13 production by liver and lymph node LECs from Brefeldin A treated mice. LECs were gated as in 3A. **(B)** Quantification of A. *P < 0.05, **P < 0.01, ***P < 0.001.

Above we demonstrated that the IL13 signaling pathway is activated in LECs of people with NASH (Figure 3) and increased IL13 gene and protein expression *in vitro* when LECs were treated with oxLDL (Figures 5D–F). We next asked if LECs in the liver and LN of mice treated with oxLDL were able to produce IL13 protein *in vivo*. To answer this question, we intravenously injected C57BL/6 mice with fluorescently labeled, oxLDL or the interferon inducing toll like receptor agonist, polyI:C (as an inflammatory control that should not induce IL13). To determine if these stimuli resulted in the production of IL13 by liver and/or lymph node (LN) LECs we directly measured production of cytokines *in vivo* 6 days post-injection (Figure 6A). Using an *in vivo* Brefeldin A assay (25, 37, 65), we found that acute stimulation with oxLDL, but not polyI:C elicited the production of IL13 by liver LECs (Figure 6B). Interestingly, while the LECs in the skin draining LN were able to take up oxLDL, they did not produce IL13 (Figure 6). These findings were confirmed using Balb/c mice that have been engineered to express YFP under the control of the IL13 promoter (IL13-YFP) (Supplementary Figure 5). These findings support our conclusions that liver LECs directly respond to oxLDL by producing IL13 and that liver LECs have a unique functional response to dietary stimulation compared to lymph node LECs.

## Discussion

The role of lymphatic vessels in normal and disrupted liver homeostasis has largely been ignored. Previous studies have demonstrated an increase in lymphatic vessel-like structures in the liver during chronic viral infection and in the setting of portal hypertension (29, 66). Others have proposed that the ascites associated with chronic liver disease may be a consequence of lymphatic dysfunction or increased lymphatic permeability (13). Furthermore, altered lymphatic function in sites peripheral to the liver has been documented in humans and animal models of obesity, infection, and hypercholesterolemia (18–20, 67). These findings seem to link liver function and lymphatic function, however even recent studies utilizing single cell RNA sequencing to evaluate liver cell populations or even specifically liver endothelial cell populations have failed to identify lymphatic endothelial cells (26, 27). This is likely due to the low frequency of lymphatic endothelial cells in normal human livers and the inability to maintain LEC viability or distinguish these populations for downstream transcriptional profiling. Thus, the precise identification of and transcriptional profile of liver lymphatic vessels in steady state and during chronic liver disease had yet to be achieved. In this study we aimed to understand the lymphatic system in the non-diseased human liver and in the setting of chronic liver disease.

We have previously developed methodology to evaluate liver LECs by flow cytometry (38). In this manuscript we demonstrate a strategy that utilized both flow cytometric sorting and single-cell mRNA sequencing to directly analyze the transcriptional profile of LECs from the liver during steady state and disease. As evident from our data (Figure 2B) our flow cytometric sorting strategy does not result in a pure population of LECs but rather an enrichment of these cells. However we do demonstrate that transcriptionally the markers *PDPN, PROX1, VEGFR3, CCL21*, and *LYVE-1*, when combined together, adequately label LECs and no other cell type in the liver expresses all of these markers. We were intrigued to find that the expansion of the LEC population in the liver was a result of active cell cycle and cell division suggesting that these cells are actively responding to accommodate the inflammation associated with disease. This expansion of LECs was a direct consequence of the increased expression of pro-proliferative and anti-apoptotic gene expression in LECs in the setting of chronic liver disease. Furthermore, LECs in diseased livers maintained high expression of the chemokine CCL21 suggesting that lymphatic vessels and lymphatic endothelial cells in particular may play an active role in immune cell recruitment, trafficking or programing during chronic liver disease.

When comparing different disease etiologies we found significant differences in the LEC transcriptional profile and pathways between NASH and HCV. Of the pathways that were differentially regulated we identified IL13 signaling as uniquely upregulated in LECs from individuals with end-stage NASH (Figure 5 and Supplementary Table 2). This pathway was interesting as IL13 has been identified as a pro-fibrogenic factor in the liver (52) as well as a factor involved in regulating lymphatic stability (35, 50). Indeed, our data points to a role for LECs in IL13 signaling through the direct stimulation of LECs with oxLDL. Our findings that IL13 is not produced by LECs either in livers from patients with HCV or in response to IFNα or polyI:C suggests that IL13 is not generally produced during inflammation. Instead, these findings suggest that IL13 production by LECs is a result of increased cholesterol, specifically oxLDL, found in the liver of patients with NASH. From these findings it is difficult to determine if release of IL13 from LECs results in either autocrine or paracrine signaling. However, our *in vitro* findings suggest that when IL13 is present, either by adding exogenous IL13 (35) or by inducing IL13 production by oxLDL, that LECs receive a signal to reduce *PROX1* expression. Loss of *PROX1* expression likely results in the decreased ability of LECs to maintain branched structures *in vitro*. Thus, we predict that while LECs receive signals to divide in order to accommodate the increased inflammation in the liver during disease, that in NASH the vessels become unstable and perhaps more permeable. These findings are intriguing as ascites is associated with chronic liver disease and may be due to increased permeability of the liver lymphatics (13). This is an important consideration when evaluating treatment options for patients with NASH compared to patients with HCV especially as NASH associated CLD is on the rise. Future studies will address whether IL13 production by LECs impacts liver specific cells such as hepatic stellate cells or is important for autocrine signaling within the LECs.

## Ethics Statement

All patients provided written and informed consent and the study was approved by the institutional review boards at the University of Colorado—Anschutz.

## Author Contributions

BT and MB designed and executed experiments, analyzed results, and drafted the manuscript. JF performed experiments, analyzed the data, and critically reviewed the manuscript. AG, KR, RF, and JH designed experiments, analyzed single cell mRNA sequencing data and critically reviewed the manuscript. MK provided samples, designed experiments, and critically reviewed the manuscript. RS analyzed immunofluorescence data and critically reviewed the manuscript. HR provided samples and insightful discussion.

### Conflict of Interest Statement

The authors declare that the research was conducted in the absence of any commercial or financial relationships that could be construed as a potential conflict of interest.

## Abbreviations

CLD: chronic liver disease
LEC: lymphatic endothelial cell
PEC: portal endothelial cell
HBV: hepatitis B virus
HCV: hepatitis C virus
PBC: primary biliary cirrhosis
NASH: non-alcoholic steatohepatitis
ALD: alcoholic liver disease
AIH: autoimmune hepatitis
PSC: primary sclerosing cholangitis
PDPN: podoplanin
IL13: Interleukin 13
MELD: model for end-stage liver disease.
