# Supplementary material for: Chronic Liver Disease in Humans Causes Expansion and Differentiation of Liver Lymphatic Endothelial Cells
Source: Front Immunol. 2019 May 15;10:1036. doi: 10.3389/fimmu.2019.01036 (PMC6530422; doi:10.3389/fimmu.2019.01036)
Supplement: Supplementary file 1 [file Data_Sheet_1.docx]

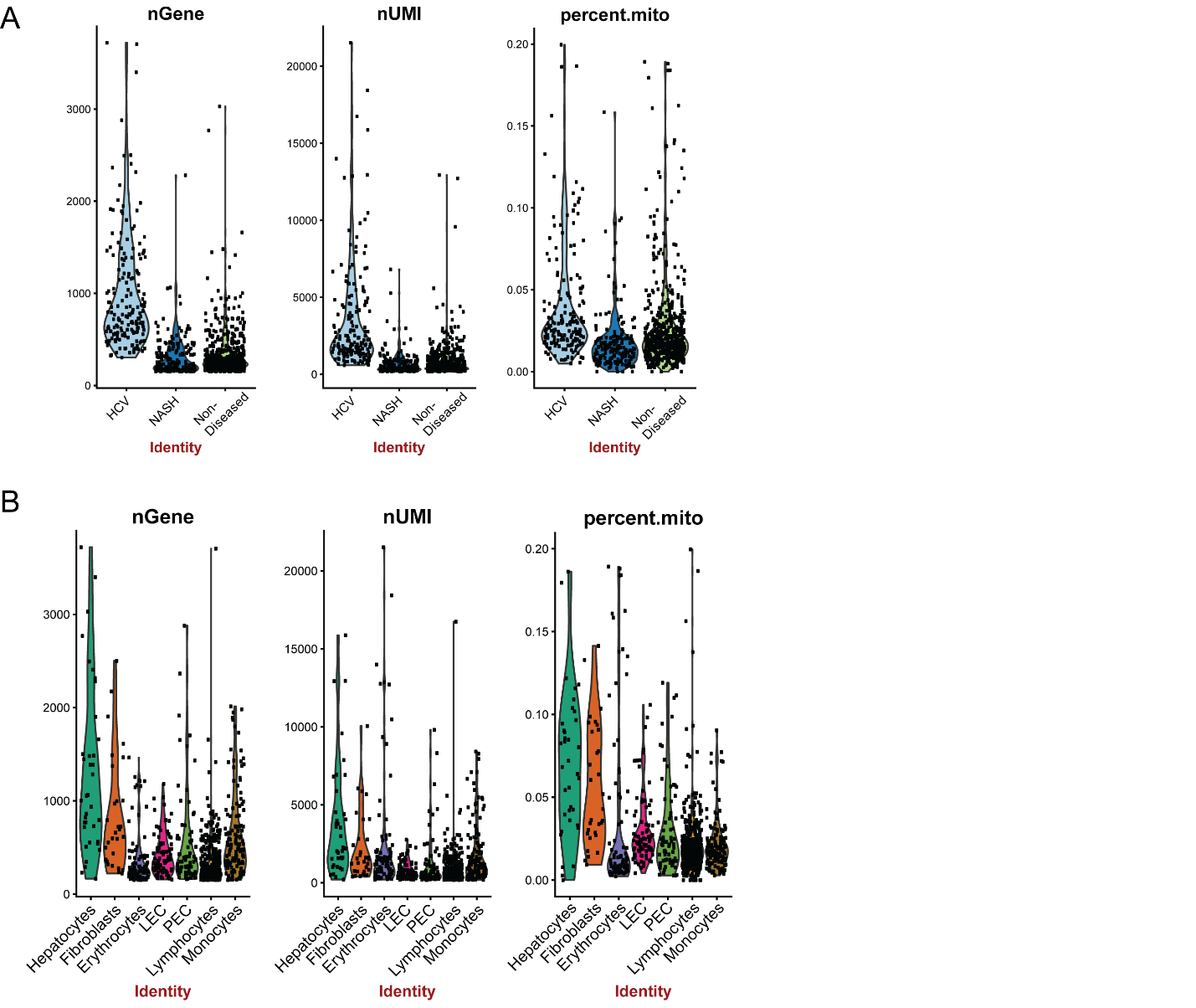


**Supplementary Figure 1.** Quality control data for single cell mRNA sequencing. Number of genes (nGene), unique molecular identifiers (nUMI) and percent mitochondrial transcripts (percent.mito) after data processing based on sample **(A)** and cell cluster **(B)**.

**
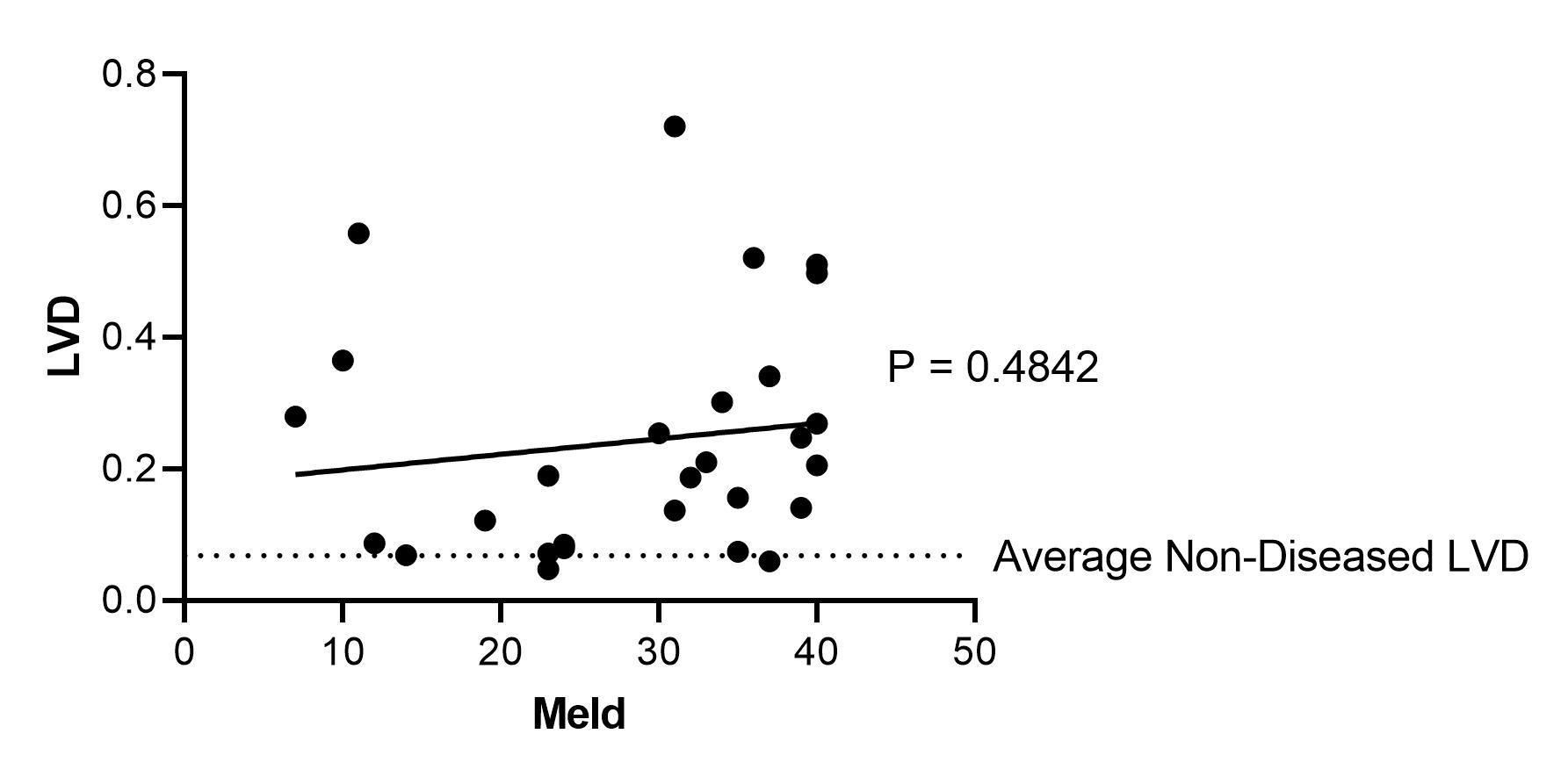
Supplementary Figure 2.** Meld score does not predict Lymphatic vessel density in the liver. All patients with chronic liver disease were plotted for MELD score and LVD. Linear regression analysis resulted in a p value of 0.4842. Multiple linear regression analysis that included BMI, Age, Race and disease etiology did not detect a significant correlation with LVD.

**
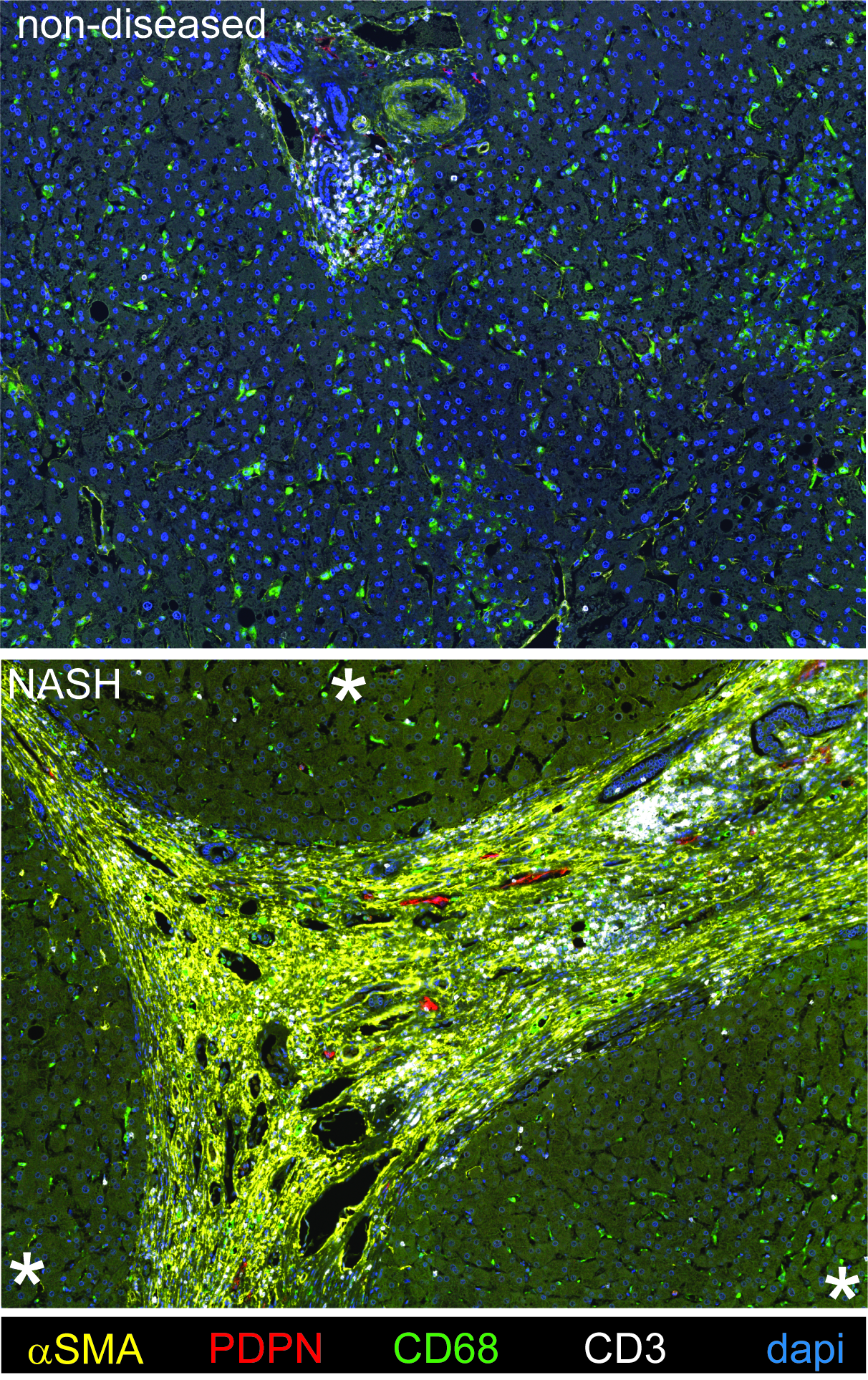
**

**Supplementary Figure 3.** Lymphatic proliferation occurs in the fibrotic areas of diseased livers. **(A)** Representative images of lymphatic vessels (Red), SMA (Yellow), Dapi (Blue) and CD68 (Green) and CD3 (White) from a non-diseased or a liver of a patient with NASH induced cirrhosis. * = regenerative nodules


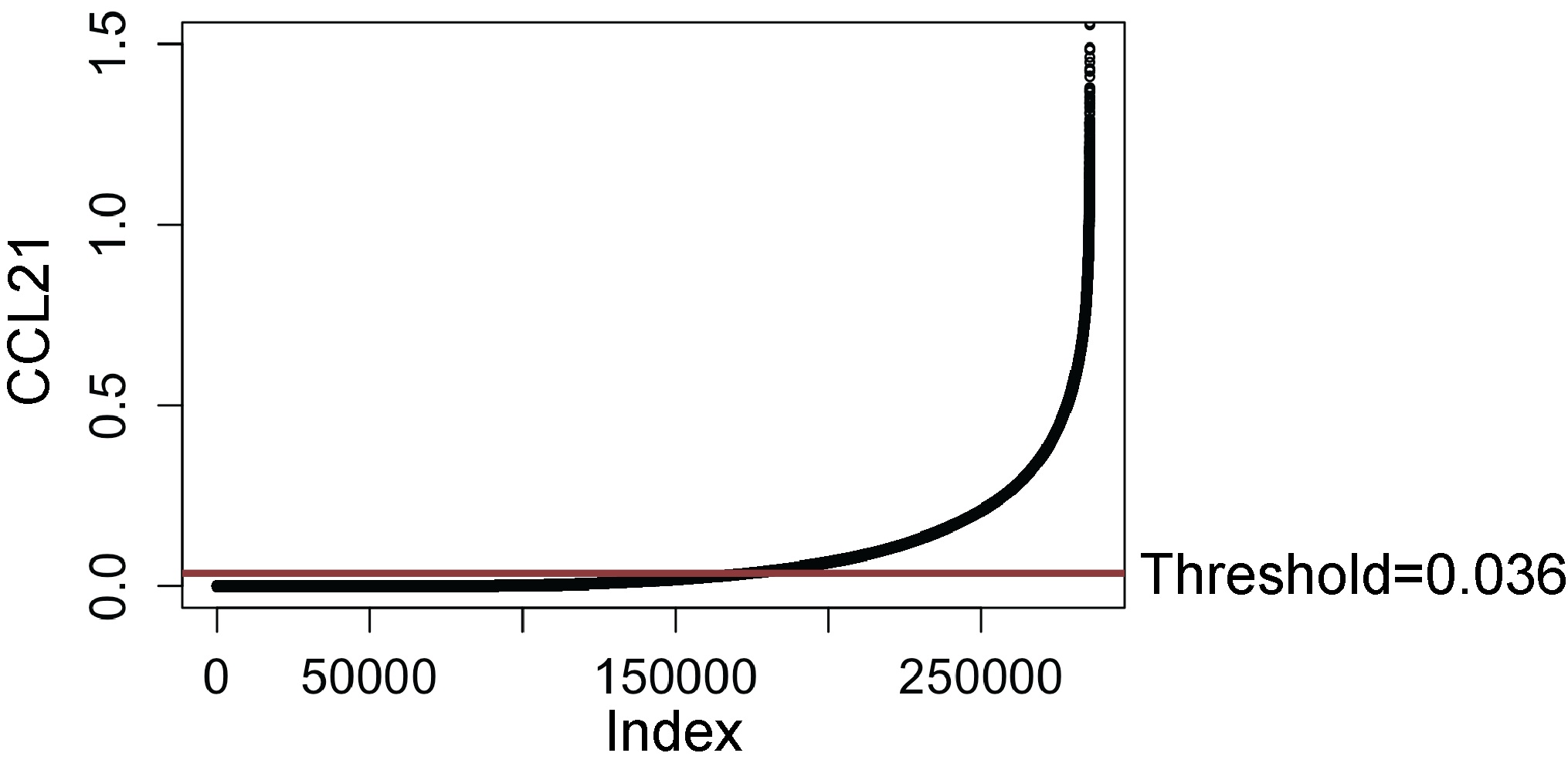


**Supplementary Figure 4.** Total values of CCL21 observed. The red line is the threshold of 0.036 to which we chose as a cut off for classifying a cell as CCL21^hi^.

**
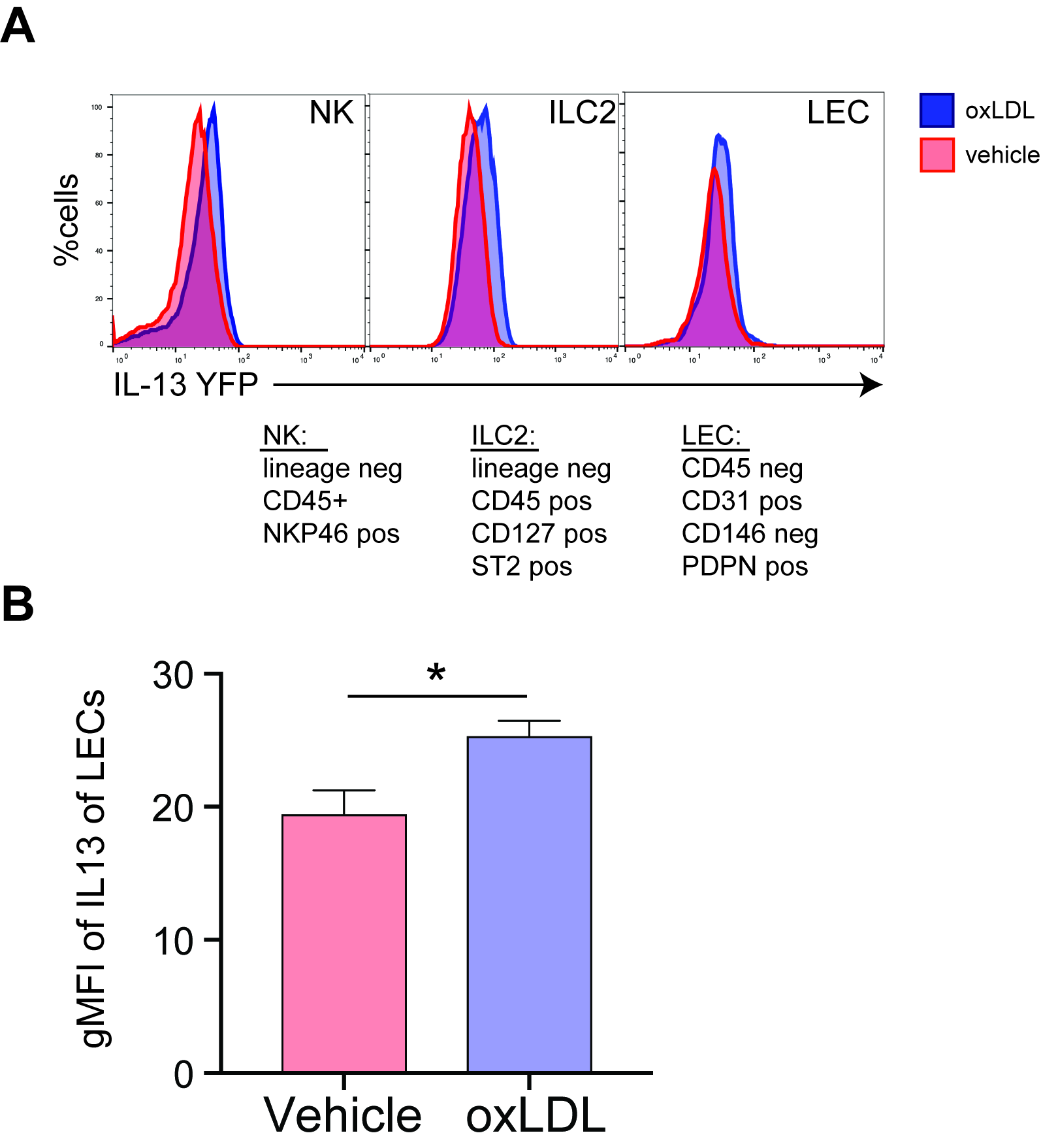
**

**Supplementary Figure 5.** Ox-LDL injection induces the expression of IL13 by LECs, NK and ILC 2 cells. **(A)** IL13-YFP (C.129S4(B6)-*IL13^tm1(YFP/creLky^*/J) were injected with 85μg of oxidized LDL. Representative flow cytometric plots of YFP expression of indicated cell types in the liver in response to vehicle (red histogram) or Ox-LDL (blue histogram) stimulation. **(B)** Quantification of geometric mean fluorescence intensity (gMFI) of YFP (IL13) in LECs. N = 3 for Vehicle (Red) and N = 6 for Ox-LDL (blue). * = P < 0.05

| **Disease Type** | **MELD** | **YOB** | **Age** | **Sex** | **BMI** | **Transplant Year** | **RACE** | **Fibrosis** | **Lymphatic Vessel Density** |
| --- | --- | --- | --- | --- | --- | --- | --- | --- | --- |
| NASH | 31 | 1949 | 60 | M | N/A | 2009 | C | 4 | 0.137171 |
| NASH | 33 | 1966 | 43 | F | 29.01 | 2009 | C | 4 | 0.210105 |
| NASH | 35 | 1967 | 47 | F | 39.01 | 2014 | Native A. | 4 | 0.074259 |
| NASH | 40 | 1956 | 58 | F | 33.24 | 2014 | Native A. | 4 | 0.511092 |
| NASH | 40 | 1961 | 55 | F | 38.29 | 2016 | C | 4 | 0.268949 |
| NASH | 39 | 1963 | 52 | M | 31.93 | 2015 | C | 4 | 0.247654 |
| NASH | 23 | 1963 | 49 | M | 32.36 | 2012 | C | 4 | 0.071811 |
| NASH | 24 | 1949 | 59 | F | 22.38 | 2008 | C | 4 | 0.079888 |
| NASH | 19 | 1950 | 58 | M | 32.81 | 2008 | C | 4 | 0.121716 |
| Alcohol | 37 | 1959 | 49 | F | 21.03 | 2008 | C | 4 | 0.059607 |
| Alcohol | 40 | 1957 | 56 | F | 30.42 | 2013 | C | 4 | 0.205809 |
| Alcohol | 36 | 1969 | 45 | M | 20.99 | 2014 | C - Hispanic | 4 | 0.520819 |
| Alcohol | 37 | 1967 | 49 | F | 21.2 | 2016 | C | 4 | 0.340921 |
| Alcohol | 32 | 1964 | 48 | F | 38.36 | 2012 | C - Hispanic | 4 | 0.186736 |
| Alcohol | 39 | 1963 | 50 | M | 25.93 | 2013 | C - Hispanic | 4 | 0.141005 |
| Alcohol | 40 | 1957 | 56 | F | 30.42 | 2013 | C | 4 | 0.4974 |
| Alcohol | 31 | 1961 | 50 | M | 22.9 | 2011 | C | 4 | 0.721293 |
| Alcohol | 24 | 1968 | 41 | F | 27.3 | 2009 | C | 4 | 0.085227 |
| Alcohol | 23 | 1959 | 49 | M | 27.12 | 2008 | C | 4 | 0.189962 |
| Alcohol | 23 | 1946 | 63 | M | 25.41 | 2009 | C - Hispanic | 4 | 0.047483 |
| HCV | 12 | 1952 | 53 | F | 20.1 | 2005 | Asian | 4 | 0.087157 |
| HCV | 10 | 1955 | 56 | M | 25.1 | 2000 | C | 4 | 0.364826 |
| HCV | 35 | 1960 | 54 | M | 30 | 2014 | C - Hispanic | 4 | 0.156042 |
| HCV | 30 | 1959 | 55 | F | 25.5 | 2015 | C - Hispanic | 4 | 0.254445 |
| HCV | 34 | 1947 | 68 | F | 25 | 2016 | C | 4 | 0.30147 |
| Wilson's | 14 | 1967 | 49 | F | 26.8 | 2017 | C | 4 | 0.068841 |
| Autoimmune | 11 | 1962 | 55 | F | 31.3 | 2017 | Native A. | 4 | 0.558306 |
| PSC | 7 | 1973 | 44 | M | 25.4 | 2018 | C | 3 | 0.279769 |
|  |  |  |  |  |  |  |  |  |  |
|  | **Cause of death** |  |  |  |  |  |  |  |  |
| **Non-Diseased** | cardiac arrest |  | 84 | F |  |  | C |  | 0.022571 |
| (From NRDI) | cardiogenic shock |  | 91 | M |  |  | C |  | 0.055031 |
|  | ALS |  | 70 | M |  |  | C |  | 0.135664 |
|  | cardiogenic shock |  | 87 | F |  |  | C |  | 0.058915 |

**Supplementary Table 1.** Demographics of patients used to determine LVD.

| **Categories** | **Diseases or Functions Annotation** | **p-Value** | **Predicted Activation State** | **Activation z-score** | **# Molecules** |
| --- | --- | --- | --- | --- | --- |
| Free Radical Scavenging | Production of superoxide | 0.0000116 | Increased | 2.425 | 6 |
| Free Radical Scavenging | Production of reactive oxygen species | 0.00118 | Increased | 2.407 | 7 |
| Free Radical Scavenging | Metabolism of reactive oxygen species | 0.00254 |  | 1.924 | 8 |
| Cell Signaling,Post-Translational Modification | Tyrosine phosphorylation of protein | 0.00116 |  | 1.413 | 5 |
| Cellular Movement | Cell movement of blood cells | 0.00313 |  | 1.193 | 11 |
| Protein Synthesis | Translation of protein | 4.36E-35 |  | 1.091 | 34 |
| Protein Degradation,Protein Synthesis | Catabolism of protein | 0.00795 |  | 1 | 10 |
| Cellular Movement | Migration of prostate cancer cell lines | 0.00314 |  | -1.091 | 5 |
| Cardiovascular System Development and Function,Cell-To-Cell Signaling and Interaction | Adhesion of endothelial cells | 0.00323 |  | -1.091 | 5 |
| Cardiovascular System Development and Function,Cell-To-Cell Signaling and Interaction | Adhesion of vascular endothelial cells | 0.00638 |  | -1.091 | 4 |
| Cell Death and Survival | Cell death of neuroblastoma cell lines | 0.000337 |  | -1.355 | 7 |
| Cell Death and Survival | Necrosis | 0.00038 |  | -1.44 | 35 |
| Cell Death and Survival | Cell death of tumor cell lines | 0.000466 |  | -1.531 | 30 |
| Infectious Diseases | Replication of RNA virus | 0.000167 |  | -1.532 | 13 |
| Cell-To-Cell Signaling and Interaction | Adhesion of tumor cell lines | 0.0066 |  | -1.558 | 8 |
| Infectious Diseases | Replication of virus | 0.0000893 |  | -1.612 | 14 |
| Cell-To-Cell Signaling and Interaction | Adhesion of leukemia cell lines | 0.00728 |  | -1.981 | 4 |
| Cell Death and Survival | Apoptosis of tumor cell lines | 0.0015 | Decreased | -2.045 | 24 |
| Cell Death and Survival | Apoptosis | 0.000041 | Decreased | -2.264 | 36 |

**Supplementary Table 2.** Table of disease functions differentially regulated between LECs from Diseased or non-diseased livers. Activated pathways are increased in diseased LECs while inhibited pathways are increased in non-diseased LECs.

| **Upstream Regulator** | **Predicted Activation State** | **Activation z-score** | **p-value of overlap** | **Target molecules in dataset** |
| --- | --- | --- | --- | --- |
| DAP3 | Activated | 2.236 | 2.36E-08 | MT-CO1,MT-CO2,MT-ND2,MT-ND3,MT-ND4L |
| TNF | Activated | 2.213 | 0.264 | DUSP1,KLF6,MT-CO2,RPS13,SOX4 |
| Jnk | Activated | 2 | 0.00409 | APP,DUSP1,KLF6,ZFP36 |
| ALKBH1 | Activated | 2 | 0.000000185 | MT-CO1,MT-CO2,MT-ND2,MT-ND4L |
| NSUN3 | Activated | 2 | 0.000000185 | MT-CO1,MT-CO2,MT-ND2,MT-ND4L |
| MAPK1 | Activated | 2 | 0.0429 | DUSP1,HBA1/HBA2,HBB,HLA-C |
| ERBB2 | Activated | 2 | 0.0614 | CDC42,ID1,JUNB,SOX4,TUBA1A |
| TGFB1 |  | 1.934 | 0.108 | ID1,JUNB,SOX4,TUBA1A,ZFP36 |
| PDGF BB |  | 1.65 | 0.0000209 | CCNL1,DUSP1,IER2,JUNB,KLF6,ZFP36 |
| HSP90B1 |  | -1.342 | 0.00000236 | CDC42,RAB13,RPL27A,RPS20,SNX3 |
| CST5 |  | -1.633 | 0.0158 | EEF1D,HNRNPH1,NR2F1,PRDX1,S100A11,SOX4 |
| TCR |  | -1.715 | 9.85E-08 | MAF,RPL17,RPL18A,RPL3,RPL4,RPL9,RPS13,RPS2,RPS23,RPS3,RPS4X,RPSA |

**Supplementary Table 3.** Table of activated or inhibited upstream regulators differentially regulated between LECs from Diseased or non-diseased livers. Activated pathways are increased in diseased LECs relative to non-diseased LECs.

| **Upstream Regulator** | **Molecule Type** | **Predicted Activation State** | **Activation z-score** | **p-value of overlap** | **Target molecules in dataset** |
| --- | --- | --- | --- | --- | --- |
| HSF1 | transcription regulator | Activated | 2.333 | 0.0000056 | CBX3,HNRNPA3,HSP90AA1,HSP90AB1,HSPA1A/HSPA1B,HSPH1,RPL22,ST13,TRA2B,UBB |
| LONP1 | peptidase | Activated | 2 | 0.000000805 | ATP5IF1,HNRNPA2B1,MT-ATP6,MT-CO2,MT-CYB,NDUFA11,S100A11,SMDT1,SOD1 |
| WISP2 | growth factor | Activated | 1.982 | 0.00307 | IGFBP7,JUN,KLF4,TFF3 |
| TP63 | transcription regulator | Activated | 1.964 | 0.0674 | CDC42,GAPDH,HES1,ID3,IGFBP7,KLF6,KRT10 |
| CLDN7 | other | Activated | 1.726 | 0.0000197 | ATP5MC1,HLA-B,IFI6,IGFBP7,LAMTOR1,MT1X,MT2A,NNMT,RPS7,SYTL2 |
| Hdac | group | Activated | 1.49 | 0.00206 | ATF3,GADD45B,JUN,KLF6,TXNIP |
| IL13 | cytokine | Activated | 1.422 | 0.024 | ATF3,CD36,FABP4,FKBP1A,MAF,MT1X,TFF3 |
| MAPK9 | kinase | Activated | 1.177 | 1.35E-08 | CAV1,GADD45B,GAPDH,HMGN2,IER2,JUNB,LMNA,LUC7L3,PPP1R15A,SEM1 |
| HIF1A | transcription regulator | Activated | 1.156 | 0.00057 | ALDOA,CAV1,CD36,GADD45B,GAPDH,GJA1,LGALS1,MIF,NPM1,NUCKS1,TCF4 |
| P38 MAPK | group | Activated | 1.118 | 0.0101 | ATF3,CD36,FABP4,JUN,MIF,RBP1,TIMP1 |
| GAPDH | enzyme | Activated | 1.114 | 0.00146 | DUSP1,GAPDH,IFI6,IFITM2 |
| Jnk | group | Inhibited | -1.046 | 0.0000101 | APP,DUSP1,GJA1,HSPA5,JUN,JUND,KLF6,TIMP1,ZFP36 |
| STAT1 | transcription regulator | Inhibited | -1.091 | 0.0317 | HLA-E,IFI6,KLF4,LY96,PSMB9 |
| TGFB1 | growth factor | Inhibited | -1.108 | 0.0193 | ALB,CD59,CRIP2,JUN,JUNB,JUND,RHOB,SOX4,TIMP1,TUBA1A,ZFP36 |
| FOXO3 | transcription regulator | Inhibited | -1.185 | 0.0019 | GADD45B,GPX1,SOD1,TXNIP,YBX1 |
| PAF1 | other | Inhibited | -1.342 | 0.000871 | ARL4A,IFITM3,KLF4,NFKBIZ,ZFP36 |
| KDM5B | transcription regulator | Inhibited | -1.342 | 0.0224 | CAV1,EIF5B,FABP5,MT1X,SAT1 |
| MAP2K1 | kinase | Inhibited | -1.432 | 0.000371 | ATF3,DUSP1,HSPA5,JUN,JUND |
| TP73 | transcription regulator | Inhibited | -1.452 | 0.0413 | HES1,HSPA1A/HSPA1B,KRT10,PNRC1,SAT1,YWHAB |
| TNF | cytokine | Inhibited | -1.714 | 0.00309 | CD59,DUSP1,HLA-B,JUN,KLF6,MT-CO2,MT-CO3,MT-CYB,PPP1R15A,PSMB9,RHOB,SAT1,SELENOP,SOX4,TIMP1,TM4SF1 |
| CDK4/6 | group | Inhibited | -1.89 | 0.0000884 | C11orf58,KRT10,MARCKS,RPL10,RPS6,SEC61B,TUBA1B |
| CCND1 | transcription regulator | Inhibited | -1.89 | 0.00205 | ATP6V0E1,C11orf58,DONSON,KRT10,MARCKS,RPL10,RPS6,SEC61B,SERF2,TUBA1B |
| ERBB2 | kinase | Inhibited | -1.89 | 0.0829 | CD59,CDC37,CDC42,HES1,JUN,JUNB,SOX4,TUBA1A |
| CEBPA | transcription regulator | Inhibited | -1.915 | 0.000845 | A2M,ALB,GJA1,HLA-B,IFI6,LGALS1,MMRN1,MT2A |
| ERK | group | Inhibited | -1.969 | 0.0749 | DUSP1,JUN,JUNB,ZFP36 |
| ALKBH1 | enzyme | Inhibited | -2 | 0.00000382 | MT-ATP6,MT-CO2,MT-CYB,MT-ND2 |
| NSUN3 | enzyme | Inhibited | -2 | 0.00000382 | MT-ATP6,MT-CO2,MT-CYB,MT-ND2 |
| IFNG | cytokine | Inhibited | -2.204 | 0.0644 | ATF3,HLA-B,HSPA1A/HSPA1B,IFI6,JUNB,KLF6,PSMB9,ST13,TXNIP |
| NUPR1 | transcription regulator | Inhibited | -2.53 | 0.245 | ATF3,CD63,HNRNPA2B1,KLF4,KLF6,MT1X,NUCKS1,PPP1R15A,SAT1,SYTL2 |
| DAP3 | other | Inhibited | -2.646 | 3.25E-10 | MT-ATP6,MT-CO2,MT-CO3,MT-CYB,MT-ND1,MT-ND2,MT-ND3 |
| PDGF BB | complex | Inhibited | -2.719 | 2.89E-08 | ATF3,CCNL1,DUSP1,IER2,JUN,JUNB,KLF6,LMNA,PPP1R15A,RHOB,ZFP36 |
| GPER1 | g-protein coupled receptor | Inhibited | -3.162 | 8.71E-10 | ATF3,CEBPD,DDIT4,DUSP1,IER2,JUN,MT1X,MT2A,PPP1R15A,ZFP36 |

**Supplementary Table 4.** Table of activated or inhibited upstream pathways in NASH compared to HCV. Activated pathways are increased in NASH while inhibited pathways are activated in HCV.
